# Incidental Diagnosis of Williams Syndrome in an Adult With Recurrent Hypercalcemia

**DOI:** 10.1210/jcemcr/luad164

**Published:** 2024-01-02

**Authors:** Seth Tersteeg, Vladimer Bakhutashvili, Margaret Crook, Heather A Ferris

**Affiliations:** Division of Endocrinology and Metabolism, University of Virginia, Charlottesville, VA 22908, USA; Division of Endocrinology and Metabolism, University of Virginia, Charlottesville, VA 22908, USA; Division of Endocrinology and Metabolism, University of Virginia, Charlottesville, VA 22908, USA; Division of Endocrinology and Metabolism, University of Virginia, Charlottesville, VA 22908, USA

**Keywords:** Williams syndrome, Williams-Beuren syndrome, recurrent hypercalcemia

## Abstract

Williams syndrome (WS) is a rare genetic disorder with multisystem involvement associated with hypercalcemia. The cause of this hypercalcemia is poorly understood and while primarily associated with WS children, it is also observed in adults. A 51-year-old woman with intellectual disability, renal insufficiency, recurrent pancreatitis, and intermittent hypercalcemia despite partial parathyroidectomy presented with hypercalcemia to 14 mg/dL (3.49 mmol/L; normal 8.6-10.5 mg/dL [2.12-2.62 mmol/L]) at routine follow-up. Laboratory testing was notable for acute-on-chronic renal failure with unremarkable vitamin D, urine calcium, and parathyroid hormone. She presented to the emergency department and was admitted. Treatment with bisphosphonates, calcitonin, and intravenous fluids decreased calcium to 9.4 mg/dL (2.35 mmol/L) and improved kidney function. She was discharged with recommendations for increased oral hydration, a low-calcium diet, and outpatient follow-up. Her phenotype was suspicious for WS, later confirmed with genetic testing. This case exemplifies both the increased risk of hypercalcemia in WS adults and the need to consider WS in hypercalcemic adults with intellectual disability. It also serves to illustrate the importance of recognizing WS features in potentially undiagnosed adults and reviews guidelines for hypercalcemia surveillance and management in WS adults.

## Introduction

Williams syndrome (WS or Williams-Beuren syndrome) (OMIM 194050), is a rare genetic disorder (1 in 7500-20 000 births) caused by a chromosome 7 microdeletion resulting in elastin deficiency with multisystem involvement, including the cardiovascular, endocrine, musculoskeletal, neurological, and gastrointestinal systems. Most people with WS have cardiovascular disease, distinctive “elfin” facial morphology, mild-to-moderate intellectual disability, and characteristic personality profile (disinhibited hypersociability, difficulties recognizing social norms, distractibility, and anxiety) [1, 2]. Because the gene region underlying WS was not identified until 1993 and a deficit of clinician familiarity with WS, many people with WS born earlier may be undiagnosed.

Hypercalcemia is a well-established pediatric complication of WS, with an estimated prevalence of 5% to 15%, and typically occurring between ages 6 and 30 months [1, 3]. It can recur in adults with WS at a higher prevalence than the general adult population. The exact mechanism of this hypercalcemia is unknown, but it is not believed to be parathyroid hormone (PTH) mediated and is generally treatment responsive [2, 4]. This case demonstrates a WS patient who went undiagnosed despite multiple clinician evaluations over numerous hypercalcemia episodes, illustrating the importance of recognizing WS features in adults due to their increased risk for various conditions and unique screening guidelines [5].

## Case Presentation

A 51-year-old woman with a history of recurrent pancreatitis and intermittent hypercalcemia presented to the emergency department (ED) with severe abdominal pain. Her medical history was significant for mild intellectual disability, hypertension, total abdominal hysterectomy with unilateral salpingo-oophorectomy, small bowel obstruction with laparotomy, diverticulosis with colon resection, nephrolithiasis, and chronic renal insufficiency. On ED presentation, she was hospitalized for pancreatitis, found to have pancreatic duct dilation and cholelithiasis without cholecystitis, and was treated with intravenous (IV) antibiotics and analgesia. At discharge, her serum calcium was normal at 8.6 mg/dL (2.15 mmol/L; normal range 8.5-10.5 mg/dL [2.12-2.62 mmol/L]) and she was discharged on her home hydrochlorothiazide (HCTZ) 25 mg daily. One month later, she presented with abdominal pain and vomiting, and was admitted for severe hypercalcemia (18.3 mg/dL; 4.57 mmol/L), hypophosphatemia, and acute kidney injury. The hypercalcemia was attributed to volume contraction, vomiting, and HCTZ use. She was treated with IV fluids, calcitonin, and zoledronate. She became hypocalcemic (7.0 mg/dL; 1.75 mmol/L) and was discharged on calcium acetate 2000 mg daily after discontinuing her HCTZ. She was readmitted the next week for pancreatitis symptoms and found to be hypercalcemic (12.2 mg/dL; 3.04 mmol/L), which resolved with IV fluids and antiemetics. She developed hypocalcemia (7.3 mg/dL; 1.82 mmol/L), and she was discharged on calcium citrate 600 mg twice daily, but did not take the calcium. She underwent endoscopic retrograde cholangiopancreatography for recurrent pancreatitis, which revealed gallstones, with pancreatic ductal stent placement shortly after discharge. One month later at her endocrinology follow-up, she was asymptomatic but her laboratory work revealed hypercalcemia (13.6 mg/dL; 3.39 mmol/L). This represented a significant increase from her calcium 2 weeks prior (7.0 mg/dL; 1.75 mmol/L), so she was advised to go to the ED. Table 1 summarizes the patient’s laboratory results and interventions during her multiple presentations for nausea/vomiting and/or abdominal pain.

**Table 1.** Summary of historic calcium results

Date	Lipase (8-78 U/L)	Admission Calcium (8.5-10.5 mg/dL)	Discharge Calcium	PTH (9-77 pg/mL) (0.95-8.17 pmol/L)	Calcium measured along with PTH	Treatment
09/01/17	263 U/L (8-78)	9.6 mg/dL	8.7 mg/dL			
6/27/20	44 U/L	17.1 mg/dL	7.1 mg/dL	21 pg/mL	14.2 mg/dL	IV fluids HCTZ discontinued
11/3/20	36 U/L	16.2 mg/dL	7.8 mg/dL	Not found- reportedly 180 pg/mL after discharge		IV fluids, calcitonin
12/22/20		9.8 mg/dL		42 pg/ml		Outpatient labs
03/14/22	147 U/L	10.1 mg/dL	8.4 mg/dL			
04/01/22	111 U/L	18.3 mg/dL	7.5 mg/dL Nadir 7.1	6 pg/mL	18.3 mg/dL	IV fluids, zolendronate, calcitonin, calcium acetate 667 mg TIDAC, HCTZ discontinued
04/14/22	1475 U/L	12.2 mg/dL	7.8 mg/dL Nadir 7.0	108 pg/mL	7.5 mg/dL	
05/05/22	106 U/L	14 mg/dL	9.2 mg/dL	3.8 pg/mL	13.6 mg/dL	IV fluids, zolendronate, calcitonin
07/26/22	2014 U/L	9.1 mg/dL	8.1 mg/dL Nadir 7.5	147 pg/mL		

Abbreviations: HCTZ, hydrochlorothiazide; IV, intravenous; PTH, parathyroid hormone; TIDAC, three times daily before meals.

Of note, 3 years prior she underwent workup for hyperparathyroidism due to recurrent hypercalcemia. A 3-mm posterior right thyroid hypodensity was discovered on computed tomography of the neck equivocal for parathyroid adenoma, but subsequent parathyroid sestamibi scan and thyroid ultrasound were unremarkable. She continued to experience episodic hypercalcemia with elevated PTH (180 pg/mL; 19.09 pmol/L [normal: 9-77 pg/mL; 0.95-8.17 pmol/L]) and underwent left upper parathyroidectomy of a visibly enlarged gland with a decrease in PTH to 97 pg/mL. Biopsy of the previously concerning right upper parathyroid revealed a normal gland, which was left intact. She had no family history of hypercalcemia or hyperparathyroidism. Her home medications included metoprolol 12.5 mg daily, omeprazole 40 mg daily, adult multivitamin (presumed to contain calcium and vitamin D of unknown dosage) daily, and a probiotic. She was a former smoker with a 10 pack-year history, consumed a dairy-free diet, and had 1 alcohol drink per week.

## Diagnostic Assessment

**Figure 1.**
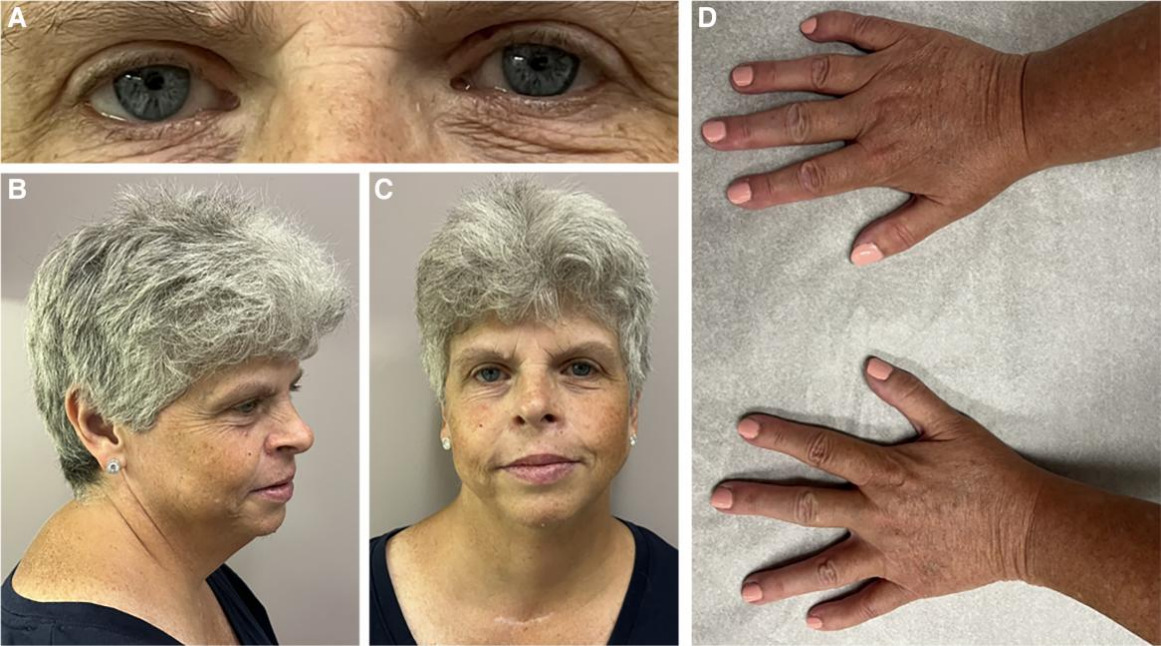
Clinical features of Williams syndrome. A, Distinct blue stellate irises. B and C, Well-healed lower neck incision, broad forehead, medial eyebrow flare, flat nasal bridge, short nose, and wide mouth. D, Bilateral fifth metatarsal clinodactyly.

On ED presentation from outpatient endocrinology, the patient’s vital signs were notable for hypertension (159/87 mm Hg), tachycardia (104 bpm), and elevated body mass index (29.17). She was well appearing on physical exam and in no acute distress. Eye examination revealed an absence of conjunctival icterus and distinct blue stellate irises (Fig. 1A). Neck examination showed a well-healed lower neck incision without thyromegaly or cervical lymphadenopathy. She had distinctive facial features, including a broad forehead, medial eyebrow flare, flat nasal bridge, short nose, small teeth, and wide mouth (Fig. 1B and 1C). Cardiopulmonary examination was normal. Abdominal examination revealed mild right lower-quadrant tenderness without rebounding or guarding. Extremities were notable for fifth metatarsal clinodactyly (Fig. 1D), low muscle bulk, and mild bilateral pitting edema to the shins. Neurological examination was unremarkable, and psychological profile revealed a friendly demeanor. Laboratory values were obtained and the results are displayed in Table 2.

**Table 2.** Laboratory results from May 5, 2022 admission

Lab value	Value	Reference range
Calcium	14 mg/dL (3.49 mmol/L) (elevated)	8.5-10.5 mg/dL (2.1-2.62 mmol/L)
Ionized calcium	6.5 mg/dL (1.62 mmol/L) (elevated)	4.4-5.4 mg/dL (1.1-1.35 mmol/L)
25-OH vitamin D	52 ng/mL (130 nmol/L) (normal)	20-80 ng/mL (50-200 nmol/L)
1,25 vitamin D	<8 pg/mL (<19.97 pmol/L) (decreased)	18-78 pg/mL (44.93-194.69 pmol/L]
Creatinine	2.0 mg/dL (176.8 μmol/L) (elevated)	0.6-1.1 mg/dL (53-97.2 μmol/L)
Parathyroid hormone	3.8 pg/mL (decreased)	9-77 pg/mL (0.95-8.17 pmol/L)
Parathyroid hormone-related peptide	0.8 pmol/L (normal)	<4.2 pmol/L
Alkaline phosphatase	85 U/L (normal)	40-150 U/L
Phosphorus	3.6 mg/dL (1.16 mmol/L) (normal)	2.6-4.7 mg/dL (0.84-1.52 mmol/L)
Urine calcium	9.8 mg (2.45 mmol)	Diet-dependent
24-h urine calcium	207 mg (51.57 mmol)	Diet-dependent

## Treatment

In the ED, the patient received zoledronate 4 mg IV and calcitonin 200 units intramuscular. Calcitonin and IV fluids were redosed on admission with improvement of calcium (14 to 9.4 mg/dL [3.49 to 2.35 mmol/L]) and creatinine (2.0 to 1.3 mg/dL [176.8 to 114.9 μmol/L]), indicating resolution of her hypercalcemia and acute kidney injury. IV fluids were tapered and she was discharged with plans to discontinue her multivitamin, ensure adequate hydration, and attend outpatient follow-up. She was also referred for a genetics workup given her unexplained recurrent hypercalcemia with phenotype suspicious for WS.

## Outcome and Follow-up

Genetics evaluated her and determined her phenotype to be consistent with WS, which was confirmed by chromosomal microarray revealing a heterozygous deletion in chromosome 7q.11.23. She has since followed-up with endocrinology and remained normocalcemic with 128 ounces (3.8 L) of fluid intake daily. Following removal of her pancreatic ductal stent, the patient experienced recurrent pancreatitis and has since had a larger stent placed and has undergone a cholecystectomy due to concern for recurrent gallstones. During each pancreatitis admission, her calcium, PTH, serum/urine protein electrophoreses, and vitamin D studies have remained normal. A systolic murmur was appreciated during one admission and echocardiogram revealed mild aortic regurgitation without aortic stenosis.

## Discussion

This patient was suspected to have WS based on her phenotype and recurrent hypercalcemia of unclear etiology. Her body composition, facial features, intellectual disability, and personality profile were consistent with the unique characteristics of WS [1]. Her medical history was also consistent, as hypertension, pancreatitis, nephrolithiasis, and chronic renal insufficiency occur more frequently in people with WS. Additionally, she had diverticulosis, which occurs at younger ages with WS than is typical, as do osteopenia/osteoporosis and menopause [2, 5].

Hypercalcemia in WS is a well-established but poorly understood phenomenon, occurring in 5% to 15% of young children and 8.7% to 11% of adults (vs ∼1% of the general population) [1, 2, 6]. Although typically secondary to hyperparathyroidism or another medical condition, the cause is often unidentified if it is not PTH dependent or 25-hydroxyvitamin D–intake dependent [7]. In this instance, hyperparathyroidism workup was unremarkable and 1,25 OH-vitamin D levels were consistently between 28 and 42 pg/mL (69.89-104.83 pmol/L; normal: 18-78 pg/mL [44.93-194.69 pmol/L]) aside from a low measurement (6.7 pg/mL; 16.72 pmol/L) during her April 1, 2022 hypercalcemia admission. Although the exact mechanisms of WS hypercalcemia have not been identified, research suggests involvement of endocrine, gastrointestinal, and renal abnormalities [8]. One proposed mechanism is the deletion of gene products involved in calcium and vitamin D regulation. The general transcription factor II-I (*GTF2I*) gene lies in the deleted WS region of chromosome 7, and deficiency is postulated to increase intestinal calcium absorption [9]. Given the dramatic increase in serum calcium in the setting of calcium supplementation, despite administration of zoledronate, it seems likely that calcium absorption was a factor in this patient. Another protein coded in this region, WS transcription factor, performs many functions, including vitamin D regulation. Further study is necessary, but WS transcription factor haploinsufficiency is theorized to result in aberrant vitamin D, and therefore calcium, homeostasis [10]. WS patients of all ages, even without clinical hypercalcemia, tend to have calcium concentrations nearer the upper limit of normal compared to non-WS individuals [8].

Symptomatic hypercalcemia in WS is often treatment responsive. Given their propensity for hypercalcemia, renal insufficiency, osteopenia, nephrolithiasis, and other metabolic conditions, it is important to understand screening guidelines specific to adult WS patients. Although there are fewer data for WS adults than children, hypercalcemia guidelines for adolescents may be applied to adults given the absence of evidence suggesting a need for alternative guidelines. WS adults are recommended to have serum calcium measurements every 1 to 2 years. For asymptomatic patients with calcium between 10 and 10.8 mg/dL (2.5-2.69 mmol/L), observation with plasma calcium surveillance every 1 to 3 months is appropriate. Levels between 10.9 and 11.5 mg/dL (2.7-2.87 mmol/L) justify further evaluation with creatinine, PTH, urine calcium:creatinine, and 25-hydroxyvitamin D measurements. Generally, the data suggest asymptomatic individuals with calcium levels less than or equal to 11.5 mg/dL (2.87 mmol/L) can be managed with increased oral fluid intake, reduced dietary calcium, and frequent monitoring. Patients with asymptomatic hypercalcemia greater than 11.5 mg/dL (2.87 mmol/L) or symptomatic hypercalcemia greater than or equal to 10.8 mg/dL (2.69 mmol/L) warrant endocrinology evaluation. In addition to these recommendations, patients may benefit from treatment with IV hydration, diuretics, bisphosphonates, glucocorticoids, and other methods generally employed in severe hypercalcemia treatment. Similar to WS children, newly diagnosed WS adults with recurrent hypercalcemia should undergo renal ultrasonography for nephrolithiasis assessment [5]. Importantly, given the increased risk for vitamin D deficiency and impaired bone mineral density, the risks/benefits of limiting oral vitamin D and/or calcium in WS patients with recurrent hypercalcemia need to be carefully considered. Intake of these should not be restricted in WS patients without hypercalcemia [2, 6].

## Learning Points

- Adults older than 35 years with WS, particularly those with mild intellectual disability, may have a missed diagnosis.
- Although hypercalcemia is frequently identified in pediatric WS patients and resolves, it can recur in adulthood.
- Adults with WS have higher rates of hypercalcemia (and other metabolic disorders) than the general population, the mechanisms of which are not fully understood, but are usually treatment responsive.
- Plasma calcium should be monitored at regular intervals in adult WS patients with a low threshold for further hypercalcemia workup and more frequent monitoring.

## Contributors

S.T., V.B., and H.A.F. were involved in the inpatient diagnosis and management of the patient, and M.C. was involved in the outpatient management of the patient. S.T. prepared the initial draft of the manuscript and S.T., V.B., H.A.F., and M.C. were all involved in the editing of the manuscript. All authors reviewed and approved the final draft.

## Data Availability

Original data generated and analyzed for this case report are included in this published article.

## Abbreviations

ED: emergency department
HCTZ: hydrochlorothiazide
IV: intravenous
PTH: parathyroid hormone
WS: Williams syndrome
